# Composite Polysaccharide Hydrogel Loaded with *Scutellaria baicalensis* Extract for Diabetic Wound Treatment

**DOI:** 10.3390/gels10090605

**Published:** 2024-09-23

**Authors:** Yumeng Zhu, Fangyan Li, Shuo Wang, Hongmei Shi, Minqian Zhao, Shaohong You, Sibo Su, Gang Cheng

**Affiliations:** Department of Pharmaceutics, School of Pharmacy, Shenyang Pharmaceutical University, No. 103 Wenhua Road, Shenyang 110016, China; zhuyumeng1231@outlook.com (Y.Z.); fylisyphu123@163.com (F.L.); w15732936967@163.com (S.W.); shm18285598793@163.com (H.S.); zmq_ling@163.com (M.Z.); ysh03182024@163.com (S.Y.); su961686@163.com (S.S.)

**Keywords:** diabetic wound, sesbania gum, traditional Chinese medicine, *Scutellaria baicalensis*, polysaccharide hydrogel

## Abstract

Diabetic wounds present significant burdens to both patients and the healthcare system due to their prolonged inflammatory phase and adverse microenvironment. Traditional Chinese medicine (TCM), particularly *Scutellaria baicalensis* extract (SE), has shown promise in wound healing. Herein, sesbania gum (SG) was oxidized and formed hydrogel with carboxymethyl chitosan (CMCS) through the imine bond. Then, SE was loaded into the hydrogel as a wound dressing (CMCS−OSG@SE hydrogel). In vitro experiments demonstrated the mechanical properties and ROS scavenging efficiency of the hydrogel, as well as the release of SE and its biocompatibility. In an vivo study, diabetic mice with *S. aureus* infection were used, and the CMCS−-OSG@SE hydrogel dressing accelerated wound healing by promoting epidermal regeneration and collagen deposition. This composite polysaccharide hydrogel loaded with SE shows great potential for diabetic wound treatment.

## 1. Introduction

Diabetic wounds seriously threaten the longevity of patients and bring significant economic and health burdens. Solving the increasing diabetic wound problem has been attractive in recent years [1,2,3]. Unlike acute wounds, diabetic wounds exhibit a prolonged inflammatory phase [4,5,6]. Therefore, an efficient anti−inflammatory effect is essential in wound dressings. Traditional Chinese medicine (TCM) can efficiently regulate inflammatory levels in diabetic wound treatment [7,8]. TCM has also been known for its advantages of having multi−component, multi−target, and multi−pathway therapeutic effects [9]. These advantages also contribute to the healing of diabetic wounds.

*Scutellaria baicalensis* extract (SE) is the extract of TCM *Scutellaria baicalensis*. Many different polyphenols have been isolated from SE, including flavonoids, which are the main active compounds in SE [10]. Among them, baicalin and baicalein are the most representative [11]. Baicalin has been proven to effectively accelerate wound healing [12]. SE was administrated orally as usual because of its various therapeutic effects, such as anti−inflammation, antipyretic, anti−bacterial, anti−hypertension, anti−allergy, and sedation properties [13]. Moreover, SE also has a long history of topical administration. In ancient China, a prescription called mei shi fang recorded that SE was used as a powder to treat herpes zoster, and SE could greatly alleviate psoriasis by regulating specific pathways associated with oxidative stress, inflammation, and cytokine signaling cascades [14]. Typically, it was reported that SE had the effect of promoting the healing of oral ulcers and periodontitis by reducing the level of inflammatory factors, regulating immune function, enhancing antioxidant capacity, and promoting the regeneration of the epithelial layer [15,16]. Furthermore, SE exhibited anti−fibrotic and anti−inflammatory effects to alleviate glaucoma by inhibiting immune pathways [17]. Therefore, SE had the potential to accelerate wound recovery.

Hydrogels were widely applied in wound treatment and management because they could provide a wet wound environment by absorbing and retaining a high quantity of water in their 3D network structure [18]. Moreover, hydrogels derived from natural components have been more popular in injured tissue therapy due to their simplicity, adaptability, and biocompatibility [19]. Sesbania gum (SG) is a natural polysaccharide polymer extracted from the seed endosperm of the leguminous plant Sesbania cannabina [20]. Carboxymethyl chitosan (CMCS) is a chitosan derivative produced by substituting a hydroxyl (−OH) functional group in glucosamine units with a carboxymethyl (−CH_2_COOH) substituent, which solves the problem of the poor solubility of chitosan [21].

**Scheme 1:**
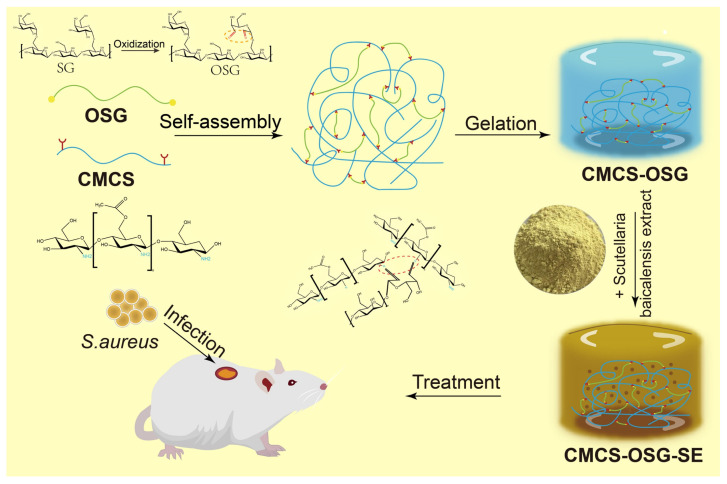
Schematic diagram of CMCS−OSG@SE hydrogel for the treatment of diabetic wounds.

In this study, SG was oxidized into OSG based on the rich hydroxyl groups of SG. Then, the composite CMCS−OSG hydrogel was fabricated by the Schiff’s base linkage between OSG and CMCS, and the SE was encapsulated into the composite hydrogel to form CMCS−OSG@SE hydrogel as a wound dressing (Scheme 1). In vitro, the characterization of CMCS−OSG@SE hydrogel was conducted by nuclear magnetic resonance spectroscopy (^1^H−NMR), Fourier transform infrared spectrometry (FT−IR), and scanning electron microscopy (SEM), and the mechanical properties of hydrogels were tested by rheology, swelling, water evaporation, water contact angle, and macroscopic healing tests. The release profile, ROS scavenging capacity, and biocompatibility of hydrogel were also assayed. In addition, the detailed effect of accelerating wound recovery was investigated in vivo.

## 2. Results and Discussion

### 2.1. Preparation and Characterization of Hydrogels

**Figure 1:**
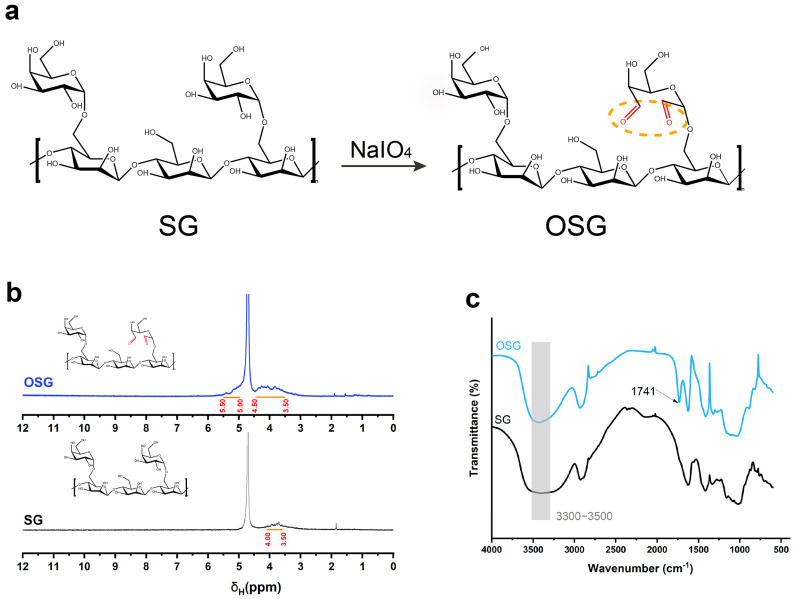
(**a**) The reaction formula of SG oxidizing to OSG. (**b**) The ^1^H−NMR spectrum of OSG and SG. (**c**) FT−IR spectra of OSG and SG.

The OSG compound was synthesized by oxidizing the hydroxyl groups of SG to form aldehyde groups (Figure 1a). In ^1^H−NMR, a broad peak at the chemical shift 3.50~4.00 ppm was present in SG, representing the hydrogen proton peak on the SG structure. In the OSG spectrum, the existence of carbonyl electronegativity increased the chemical shift of the hydrogen proton on the SG sugar ring, and a new broad peak belonging to the hemiacetal group appeared between the chemical shift of 5.00~5.50 ppm in the OSG (Figure 1b). In addition, in the FT−IR results, the wide absorption peak from 3500 cm^−1^ to 3300 cm^−1^ represented the expansion and vibration peak of the hydroxyl group. The peak strength was weakened after oxidation, indicating that the oxidizing modification replaced the hydroxyl group in the structure of SG. The peak at 1741 cm^−1^ in OSG (Figure 1c) represented the carbonyl stretching vibration [22].

**Figure 2:**
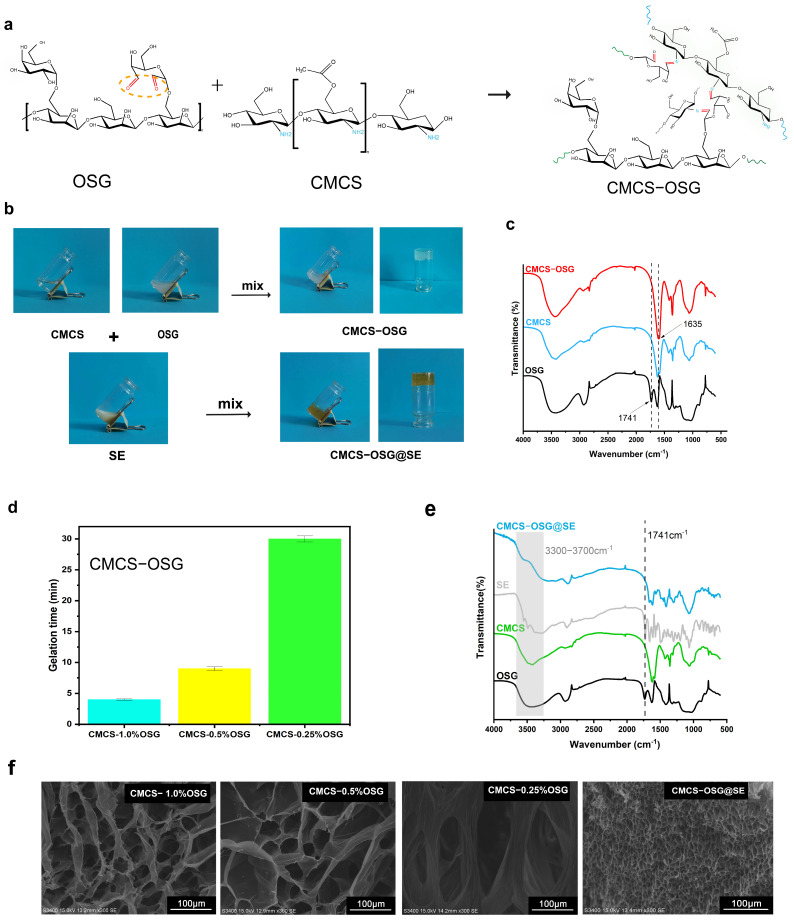
(**a**) The reaction formula of gelling theory. (**b**) The gel–sol transition of CMCS−OSG and CMCS−OSG@SE hydrogels. (**c**) FT−IR spectra of CMCS−OSG, CMCS, and OSG. (**d**) Gelation times of CMCS−OSG hydrogels with different amounts of OSG. (**e**) FT−IR spectra of CMCS−OSG@SE, SE, CMCS, and OSG. (**f**) SEM image of CMCS−OSG and CMCS−OSG@SE hydrogels.

The reaction formula of the gelling theory is listed in Figure 2a. In CMCS−OSG hydrogel, a peak at 1635 cm^−1^ replaced the carbonyl stretching vibration peak at 1741 cm^−1^, which represented the imine bond stretching vibration (Figure 2c), and in the CMCS−OSG@SE FT−IR results (Figure 2e), the carbonyl stretching vibration peak also disappeared. These results verified that the formation of hydrogels depended on the Schiff base reaction [23]. Furthermore, the absorption peak from 3300 to 3700 cm^−1^ in CMCS−OSG@SE was consistent with SE, indicating that the presence of SE changed the hydrogen bond interaction in the hydrogel. In Figure 2b, CMCS−OSG and CMCS−OSG@SE hydrogels both had a consistent, jelly−like appearance. The CMCS−OSG@SE hydrogel had a tawny color, while the CMCS−OSG hydrogel was transparent.

The gelation time of CMCS−OSG was dependent on the cross−linking formed by the reversible imine bond. The whole gelation times of CMCS−OSG (1–0.25%) hydrogels were in a range of 4–30 min (Figure 2d). With the decrease in OSG, the gelation time increased. The gelation time of CMCS−OSG significantly increased to 30 ± 0.5 when the content of OSG was 0.25%. Therefore, the content of OSG in hydrogel should be greater than 0.5% to provide sufficient carbonyl groups for the rapid cross−linking of the hydrogel. The CMCS−OSG@SE hydrogel was only 42 s. The SE extract contains ingredients such as dove and flavonoids. These ingredients promote the formation of imine bonds, accelerating the cross−linking of hydrogel. SEM further showed the porous structure inherent to CMCS−OSG and CMCS−OSG@SE hydrogels (Figure 2f). With the increase in OSG, the pore size of hydrogel was smaller. The mean diameters of CMCS−0.25% OSG, CMCS−0.5% OSG, and CMCS−1.0% OSG hydrogels were 143.85 ± 21.76 μm, 58.39 ± 24.40 μm, and 45.39 ± 8.08 μm. Increasing the OSG content enhanced the cross−linking in the hydrogel by forming more imine bonds, resulting in a reduction in pore size. The mean diameter in CMCS−OSG@SE hydrogel was 3.67 ± 0.56 μm, illustrating that SE participated in the cross−linking process, contributing to a tighter network. This result was consistent with the gelation time.

### 2.2. Mechanical Properties of Hydrogels

**Figure 3:**
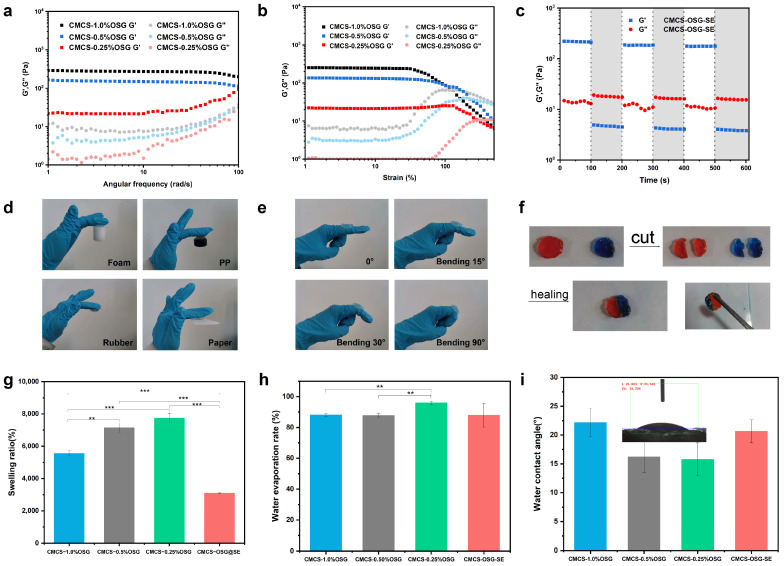
(**a**) Rheological frequency–sweep curves of CMCS−OSG hydrogels. (**b**) Rheological strain–sweep curves of CMCS−OSG hydrogels. (**c**) Rheological time–sweep curves of CMCS−OSG@SE hydrogels after three cycles (1–300%) are applied. (**d**) Photographs of the CMCS−OSG hydrogel clinging to different materials. (**e**) Photographs of the CMCS−OSG hydrogel clinging to the elbow joint under different angles. (**f**) Macroscopic self−healing test of the CMCS−OSG hydrogel. (Methyl orange and methylene blue were used as dyes). (**g**) Swelling ratio of CMCS−OSG and CMCS−OSG@SE hydrogels. (**h**) Water evaporation rate of CMCS−OSG and CMCS−OSG@SE hydrogels. (**i**) Water contact angle of CMCS−OSG and CMCS−OSG@SE hydrogels. (** *p* < 0.01, and *** *p* < 0.001).

Oscillatory rheology was used to investigate the physical properties of CMCS−OSG and CMCS−OSG@SE hydrogels. To characterize the rheological properties of the CMCS−OSG and CMCS−OSG@SE hydrogels, the changing profiles of the storage modulus (G′) and loss modulus (G″) were studied under various assays [24]. After the successful fabrication of the hydrogel, it was observed that the G′ was higher than the G″ in the CMCS−OSG hydrogel samples. The results depicted that the G′ of the CMCS−OSG hydrogel was significantly higher than that of the CMCS−OSG hydrogel with low OSG (Figure 3a). An increased OSG content can enhance the cross−linking density, resulting in a more compact hydrogel structure that improves resistance to external forces (high G′ and G″). In the strain sweep mode, the sol–gel transform points of CMCS−1.0% OSG, CMCS−0.5% OSG, and CMCS−0.25% OSG hydrogels were 130%, 231%, and 317% strain, respectively (Figure 3b). This is because increased cross−linking leads to a more compact network structure, which enhances tensile strength but reduces toughness (a low sol–gel transform point). The time sweep mode results of CMCS−OSG@SE hydrogel showed that G′ was greater than G″ under low strain (1% strain) conditions, while G″ was greater than G′ under high strain (300% strain) conditions, indicating a sol–gel transition. The gel–sol collapse of the CMCS−OSG@SE hydrogel at the large strain of 300% could recover at a low strain of 1% during three cycles, indicating the superb self−healing property of CMCS−OSG@SE hydrogels (Figure 3c). The cross−linking of the hydrogel depended on the formation of reversible imine bonds. When exposed to external forces, the imine and hydrogen bonds that broke internally spontaneously reformed, enabling the hydrogel to regain its cross−linking structure and exhibit self−healing properties.

Hydrogels with self−healing properties can extend their lifespan and decrease consumption [25,26]. In addition, hydrogel wound dressings should have proper physical adhesion and stretchability to completely cover the wound, thereby providing a closed and moist microenvironment that is conducive to promoting wound healing [27]. As shown in Figure 3d, the CMCS−OSG hydrogel clung to various material surfaces (foam, PP, rubber, and paper). Notably, when the hydrogel clung to the skin and elbow joint under different angles (Figure 3e), both the hydrogel and the joint could twist casually. These results indicate that the hydrogel would not interfere with body motion during application. In Figure 3f, the two hydrogels were first cut into four pieces, and then the two halves of them were put together. Without any external forces, the two halves clung together to reform a complete gel. The swelling ratios of hydrogels are shown in Figure 3g. The swelling ratios of CMCS−1.0% OSG, CMCS−0.5% OSG, CMCS−0.25% OSG, and CMCS−OSG@SE were 5560 ± 150%, 7146 ± 282%, 7734 ± 307% and 3095 ± 47%. The cross−linking density increased with the increase in OSG in the hydrogel, which stabilized the network structure and resulted in a lower swelling ratio.

Hydrogels effectively retain water around wounds, which helps speed up the healing process [28]. In addition, the water evaporation rate of CMCS−OSG hydrogels showed significant differences with the increasing content of OSG. The water evaporation rates of CMCS−1.0% OSG, CMCS−0.5% OSG, and CMCS−0.25% OSG hydrogels were 88 ± 1%, 88 ± 1% and 96 ± 1%, respectively, and the CMCS−OSG@SE group (88 ± 7%) showed no significant differences compared to the other three groups (Figure 3h), which indicated that hydrogels could contain proper water retention properties when the content of OSG exceeded 0.5%. This was related to the previously discussed cross−linking density of the hydrogel. When the OSG exceeded 0.5%, it offered an adequate number of imine bond sites, facilitating rapid cross−linking and forming a dense network capable of retaining more moisture. The water contact angle is an important property in evaluating the hydrophilicity of hydrogels [29]. The contact angle decreases as the amount of OSG decreases (the water contact angles were 22.2°, 16.2°, and 15.8° at contents of 1%, 0.5%, and 0.25% OSG, respectively), and the contact angle of the CMCS−OSG@SE hydrogel was 20.6°. All the water contact angles were <90° (Figure 3i), indicating the excellent hydrophilicity of the hydrogel [30]. These mechanical properties allowed the hydrogel dressings to maintain a moist environment for the wound and provide a physical barrier without affecting movement while continuously releasing herbal extracts to accelerate wound healing.

### 2.3. In Vitro Release and ROS Scavenging Efficiency

**Figure 4:**
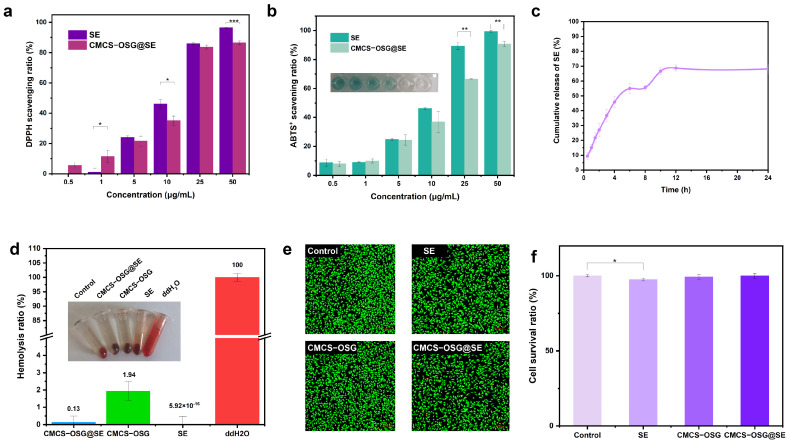
(**a**) ROS scavenging capacity of SE and CMCS−OSG@SE hydrogel for DPPH. (**b**) ROS scavenging capacity of SE and CMCS−OSG@SE hydrogel for ABTS+. (**c**) Cumulative release profile of SE from CMCS−OSG@SE hydrogel in PBS. (**d**) Hemolysis ratio of SE and hydrogels. (**e**) Fluorescence of living cells in the control, SE, CMCS−OSG, and CMCS−OSG@SE hydrogels groups for 24 h. (**f**) L929 cell survival ratio after incubating for 24 h. (* *p* < 0.05, ** *p* < 0.01, and *** *p* < 0.001).

An excessive ROS expression at diabetic wound sites can cause severe inflammation and disrupt normal physiological processes [31,32,33]. The total ROS scavenging ability of SE and CMCS−OSG@SE hydrogel was investigated by ABTS+ and DPPH methods in vitro. As shown in Figure 4a,b, both the SE and CMCS−OSG@SE hydrogel could eliminate the ABTS+ free radical, and the scavenging ability (15.5 ± 0.5%–80.7 ± 6.0%) displayed a concentration−dependent performance (0.5 μg/mL–50 μg/mL). Meanwhile, DPPH scavenging results also revealed a prominent free radical scavenging ability of 96.4 ± 0.4% at an SE concentration of 50 μg/mL, while the free radical scavenging ability was 86.5 ± 1.1% at a CMCS−OSG@SE concentration of 50 μg/mL. This may be related to the slow release of SE from the CMCS−OSG@SE hydrogel. In the PBS buffer, the cumulative release of SE reached saturation at 12 h, with 68.8 ± 2.1% (Figure 4c).

### 2.4. Biocompatibility of Hydrogels

**Figure 5:**
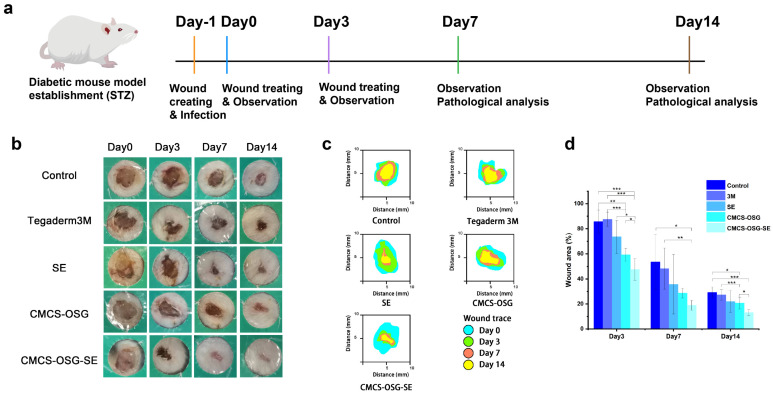
(**a**) Mechanism diagram of the mice wound treatment process. (**b**) Representative photos of wounds treated in the non−treatment group (control), 3M film group, SE group, CMCS−OSG hydrogel group, and CMCS−OSG@SE hydrogel group on days 0, 3, 7, and 14. (**c**) The wound area for treatment in the control group, the 3M, the SE, the CMCS−OSG hydrogel, and the CMCS−OSG@SE hydrogel on days 0, 3, 7, and 14 are shown. (**d**) On days 0, 3, 7, and 14, the wound area percentage of the control group, the 3M, the SE, the CMCS−OSG hydrogel, and the CMCS−OSG@SE hydrogel are shown (n = 5). (* *p* < 0.05, ** *p* < 0.01, and *** *p* < 0.001).

Good biocompatibility is essential for hydrogels to be effective as wound−healing dressings [34,35]. The hemolysis test results are shown in Figure 5d. The hemolysis rates of SE, CMCS−OSG, and CMCS−OSG@SE hydrogels were only 5.29 × 10^−16^ ± 0.48%, 1.94 ± 0.56%, and 0.13 ± 0.37% at a concentration of 1 mg/mL, respectively. The cytocompatibility of hydrogels was evaluated by L929 fibroblasts. The results are shown in Figure 4e,f. After 24 h of cultivation, the cell survival ratios of all samples were above 95%. A significant difference was observed between the SE group and the control group, indicating that SE might inhibit cell spread to a certain extent. In addition, live and dead cell staining experiments showed no significant difference in L929 cell viability.

### 2.5. Wound Healing Ability of Hydrogels

*S. aureus* is the most common microorganism in diabetic foot infections [36]. The full−thickness skin defect complicated by the bacterial infection diabetic wound model was used to further evaluate the effectiveness of the hydrogels in accelerating wound healing in vivo [37,38]. The experimental procedures are shown in Figure 5a. The non−treated group was set as a control group, and the wound site was treated with 3M, an SE water solution, blank CMCS−OSG hydrogel, and CMCS−OSG@SE hydrogel, respectively. Figure 5b shows the representative photos of the wound site. The dry crust was obviously present in the control and 3M groups due to dehydration, while the wound healing process was significantly accelerated in the SE, CMCS−OSG, and CMCS−OSG@SE hydrogel groups. Wound recovery was notably rapid within the first 7 days. Compared with other groups, the CMCS−OSG@SE hydrogel group had an obviously better healing process. Specifically, the wound area of different treatment groups was calculated and is shown in Figure 5c,d. At the beginning of wound healing, severe inflammation occurred at the wound site, resulting in a large amount of ROS. The wound areas of SE, CMCS−OSG, and CMCS−OSG@SE hydrogels were smaller than the other two groups on day 3, which showed better anti−inflammation activities and wound healing properties. Among them, the wound area of the CMCS−OSG@SE hydrogel group was significantly reduced to 47.50 ± 8.57%, while the wound area of the control group was 85.70 ± 9.23%. On day 7, the wound area of the CMCS−OSG@SE hydrogel treatment group (18.92 ± 3.88%) was smaller than that of the control group (53.55 ± 21.87%). However, the low residue and burst release of SE groups at the wound site slowed down the wound healing process (35.70 ± 23.75%). The wound area treated with the Tegaderm 3M group showed poor closure, which might be due to the tight adhesion and poor absorbance of the membrane preventing tissue regeneration. On day 14, the wound site of the CMCS−OSG@SE hydrogel group had almost recovered completely, and the wound site of the control group still had a wound area of 29.24 ± 4.29%. At the same time, the lower promoting effects of the 3M and CMCS−OSG hydrogel on wound healing were shown. Therefore, the CMCS−OSG@SE hydrogel could absorb wound exudation, maintain a moist environment, promote wound closure, and finally accelerate wound healing.

**Figure 6:**
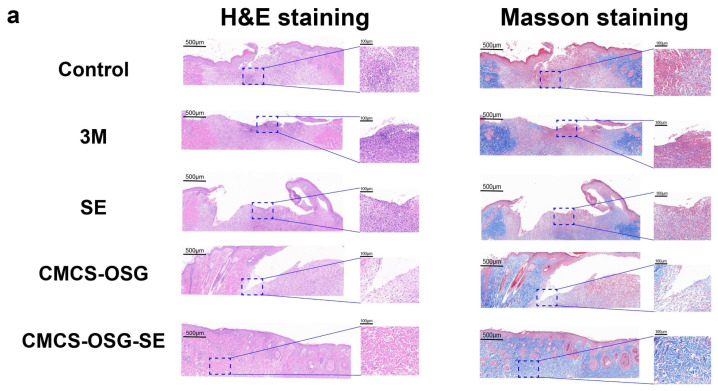

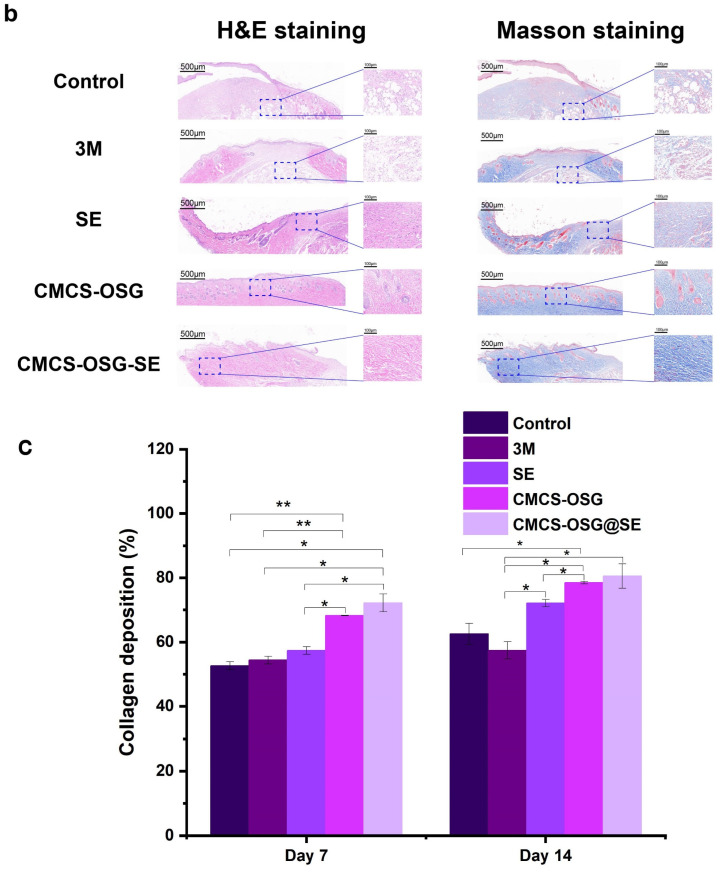
(**a**) H&E staining and Masson staining images of wound tissue with no treatment (control) or the 3M, SE, CMCS−OSG hydrogel, and CMCS−OSG@SE hydrogel treatments on day 7 and (**b**) day 14. (**c**) Collagen deposition in wound tissue for the different treatment groups on day 7 and day 14. (* *p* < 0.05 and ** *p* < 0.01).

The skin tissues on day 7 and day 14 in the wound site were collected and assessed by H&E and Masson staining pathological analysis (Figure 6a,b). The results showed that significant inflammatory cell infiltrations were presented in the control and 3M group, indicating the inflammation caused by bacterial infection and inflammatory cell migration on day 7. There were still large areas without new granulation tissue in the SE and CMCS−OSG hydrogel dressing, and the granulation tissue was thinner than that of the CMCS−OSG@SE hydrogel group. On day 14, the results (Figure 6c) showed that the skin tissue of the control and 3M groups retained segmental defects, while the SE, CMCS−OSG, and CMCS−OSG@SE hydrogels groups exhibited basic epidermal regeneration. Moreover, compared with other groups, the granulation of tissue in the CMCS−OSG@SE hydrogel tended to thicken. This result suggests that the CMCS−OSG@SE hydrogel can significantly promote the formation of granulation tissue. In Masson staining images of wound sites, there was more intense collage fiber in the CMCS−OSG@SE hydrogel group compared with the other groups (72.14 ± 1.14% in the SE group, 78.51 ± 0.42% in the CMCS−OSG hydrogel group and 80.54 ± 3.78% in the CMCS−OSG@SE hydrogel group). These pathological studies also confirm the ability of the CMCS−OSG@SE hydrogel to promote wound healing.

To better evaluate the efficiency of the CMCS−OSG@SE hydrogel, a comparison of the efficiencies of the current hydrogel with the most recently published hydrogels is listed in Table 1. All the hydrogels listed in Table 1 were formed based on chitosan and chitosan derivatives.

## 3. Conclusions

In conclusion, CMCS−OSG@SE hydrogel wound dressing was fabricated and screened. The eventual hydrogel possessed great mechanical properties and excellent self−healing. Meanwhile, the CMCS−OSG@SE hydrogel exhibited an excellent ROS scavenging capacity. Moreover, no hemolysis or cytotoxicity of the CMCS−OSG@SE hydrogel were found. It was shown that the CMCS−OSG@SE hydrogel had good biocompatibility and application values. The wound healing study in vivo suggested that the CMCS−OSG@SE hydrogel enhanced the therapeutic effect on the diabetic wound by promoting epidermal regeneration and collagen deposition. In a word, CMCS−OSG@SE hydrogel dressing is a promising wound dressing for diabetic wounds.

## 4. Materials and Methods

### 4.1. Materials

Carboxymethyl chitosan (CMCS) and streptozocin (STZ) were purchased from Shanghai Macklin Biochemical Technology Co., Ltd. (Shanghai, China). Sesbania gum (SG) was obtained from Shanghai karmachem Co., Ltd. (Shanghai, China). SE was purchased by Xi’an Liping Biotechnology Co., Ltd. (Xi’an, China). Male Kunming (KM) mice were supplied by Liaoning Changsheng Biotechnology Co., Ltd. (Shenyang, China). All other reagents were of analytical grade.

### 4.2. Synthesis of OSG

SG (1 g) was stirred to dissolve completely in 100 mL of deionized water. Subsequently, NaIO4 (2 g) was added to the SG solution. The reaction was carried out for 12 h at 25 °C water bath. Ethylene glycol (1 mL) was added to quench the reaction. Then, the dialysis was performed using a dialysis bag with a molecular weight cut−off (MWCO) of 3.5 kDa for 2 days. The final product was obtained by lyophilization.

### 4.3. Preparation of CMCS−OSG@SE Hydrogel

The obtained OSG and CMCS were weighed, dissolved, and swelled overnight at a concentration of 2% (*w*/*v*). Meanwhile, the SE was dissolved in deionized water at a concentration of 4% (*w*/*v*). Then, SE was mixed with CMCS and OSG to prepare the CMCS−OSG@SE hydrogel. The prepared CMCS−OSG hydrogels were the blank hydrogels without SE. The gelation times of the different hydrogels were measured using the inversion test [44].

### 4.4. Characterization

The synthesized OSG sample was analyzed using ^1^H−NMR with D_2_O as the solvent. Then, FT−IR was employed to characterize the chemical structure of the samples in the range of 4000 to 500 cm^−1^. The swelling ratios of hydrogels were examined by the weight method. Briefly, the dry weight (Wd) of hydrogel samples were obtained by lyophilization and immersed into a 10 mL PBS solution. The weight (Wt) of the hydrogel was measured after 24 h, and the swelling rate was obtained by the following formula:

Swelling rate=(Wt−Wd)/Wd×100%

In addition, the prepared hydrogel was exposed to room temperature, and the water evaporation rate was measured by the weight (We) at 24 h and the initial weight (W0) of the hydrogel.

Water evaporation=(W0−We)/(W0−Wd)×100%

where Wd represents the dry weight of the hydrogel. The micro−morphology of hydrogels was observed by SEM (Hitachi S−3400N, Tokyo, Japan) with lyophilized. The water contact angles were calculated using a contact angle analyzer SDC−100 (SINDIN, Dongwan, China) with three measurements at different surface sites.

### 4.5. Rheological Test

The rheological properties of hydrogels were assessed using a rheometer (AR2000ex, TA Instruments, Detragized State, Newcastle, DE, USA) with a 20 mm diameter parallel plate. A frequency sweep was conducted by setting a strain of 1% and adjusting the angular frequency from 1 to 100 Hz. A strain sweep was performed by setting an angular frequency of 1 Hz and adjusting the strain from 1% to 500%. A time sweep was conducted at an angular frequency of 1 Hz, with the strain varying from 1% to 300% over 3 cycles.

### 4.6. In Vitro SE Release in CMCS−OSG@SE Hydrogel

The CMCS−OSG@SE hydrogel (2 mL) was loaded into a dialysis bag and placed into a water bath shaker at 37 °C, 100 rpm. Then, a 1 mL sample was removed at preset interval times, and the fresh medium was added as a supplement. A UV–vis spectroscopy measurement was employed to determine the release of SE in the Supplementary Materials.

### 4.7. In Vitro ROS Scavenging Efficiency of SE and CMCS−OSG@SE Hydrogel

The ROS scavenging efficiency was assessed by the DPPH and ABTS+ kits. The DPPH and ABTS+ scavenging ratios of SE and the CMCS−OSG@SE hydrogel were calculated according to the manufacturer’s specification. In the DPPH method, the diluted SE and CMCS−OSG@SE hydrogel samples were added to the DPPH ethanol work solution (0.1 mM) and incubated. The DPPH scavenging ratio was calculated by the absorption of the corresponding sample at 517 nm. Similarly, in the ABTS+ method, the diluted SE and CMCS−OSG@SE samples were mixed with the ABTS+ working solution, and the scavenging ratio was determined by measuring the absorbance at 734 nm.

### 4.8. Biocompatibility Assay of Hydrogel

The hemocompatibility of SE, CMCS−OSG, and CMCS−OSG@SE hydrogels was investigated using fresh mice blood. Red blood cells were collected by centrifuging fresh mouse blood and washing them three times with saline. Then, the 2% red blood cell suspension was prepared. The samples were diluted to a corresponding concentration with saline to obtain sample suspensions. After incubating the sample suspension with a red cell suspension, the absorbance of the medium was determined by a microplate reader at 545 nm.

The cytocompatibility of SE, CMCS−OSG, and CMCS−OSG@SE hydrogels was assessed using the Cell Counting Kit−8 (CCK−8) method. The samples were sterilized by ultraviolet irradiation for 30 min. Sample extractions (1 mg/mL) were added to the medium and immersed in a 37 °C, 5% CO_2_ incubator. After 24 h of extraction, 1 mg/mL of the sample extractions was obtained by filtering (0.22 μm filter) and sterilizing. Then, the sample extractions were diluted to a working concentration by the medium. In the meantime, L929 cells were seeded into 96−well plates at a density of 6 × 10^3^ per well and incubated for 12 h. Then, the medium was replaced with the sample extractions (100 μL). Cell viability was assessed at 24 h by confocal laser scanning microscopy (CLSM) (ZEIS, LSM880, Oberkochen, Germany).

### 4.9. In Vivo Wound Healing Assay

All animal experiments were conducted following the National Research Council’s Guide for the Care and Use of Laboratory Animals (2011), and were approved by the Ethical Committee of Shengyang Pharmaceutical University (SYPU-IACUC-C2017-4-28-205). The STZ at a dose of 75 mg/kg was injected into Kunming mice for 5 days continuously. Then, the mice with glucose levels exceeding 16.7 mmol/L were selected and fed for an additional 5 days. A diabetic mouse model was considered successful when the fasting glucose level remained above 16.7 mmol/L. After 1 day of fasting, the full−thickness skin defect (diameter 8 mm) developed on the mice’s back and infected with *S. aureus*. The wounds were treated daily using various methods: (1) control group, (2) 3M group, (3) SE group, (4) CMCS−OSG group, and (5) CMCS−OSG@SE group. After 3, 7, and 14 days, wounds were photographed regularly. Image J software (https://imagej.net) was utilized for wound area measurement, and the percentage of wound healing was calculated. Finally, on day 7 and day 14, tissue samples from the wounds were collected, and detailed wound healing was investigated by hematoxylin and eosin staining (H&E) and Masson staining.

### 4.10. Statistical Analysis

All experiments were performed in parallel at least 3 times, and the data were presented as the mean ± SD. A statistically significant difference is shown as * *p* < 0.05, ** *p* < 0.01, and *** *p* < 0.001.

## Figures and Tables

**Table 1:** Comparison of the efficiencies of the current hydrogel with the most recently published hydrogels (chitosan−based).

Hydrogel	Rheology	Adhesion	Self−Healing	ROS Scavenging	Wound Treatment
CPN−Ga−4.1% [39]	√	√	×	×	√
QCPN@PDA [40]	×	×	×	√	√
CS/PUL [41]	√	√	×	×	×
Gel2 [42]	√	×	√	×	√
CS−RH [43]	√	×	×	×	√
Current work (CMCS−OSG@SE)	√	√	√	√	√

(The symbol “√” denoted that studies had researched the effect. The symbol “×” denoted that studies hadn’t done the assay).

## Data Availability

The original contributions presented in the study are included in the article/Supplementary Materials, further inquiries can be directed to the corresponding author.

## Supplementary Materials

The following supporting information can be downloaded at https://www.mdpi.com/article/10.3390/gels10090605/s1, Figure S1. UV-Vis full wavelength scanning of SE and blank hydrogel. Figure S2. The standard curve of SE. Table S1. Instrument precision results. Table S2. Method precision results. Table S3. Method accuracy results.
