# Supplementary material for: Composite Polysaccharide Hydrogel Loaded with Scutellaria baicalensis Extract for Diabetic Wound Treatment
Source: Gels. 2024 Sep 23;10(9):605. doi: 10.3390/gels10090605 (PMC11431221; doi:10.3390/gels10090605)
Supplement: Supplementary file 1 [file gels-10-00605-s001.zip › gels-3190208-supplementary.pdf]

gels

MDPI

The UV–vis spectroscopy method for determining the release of SE from CMCS-OSG-SE hydrogel.

Method specificity:

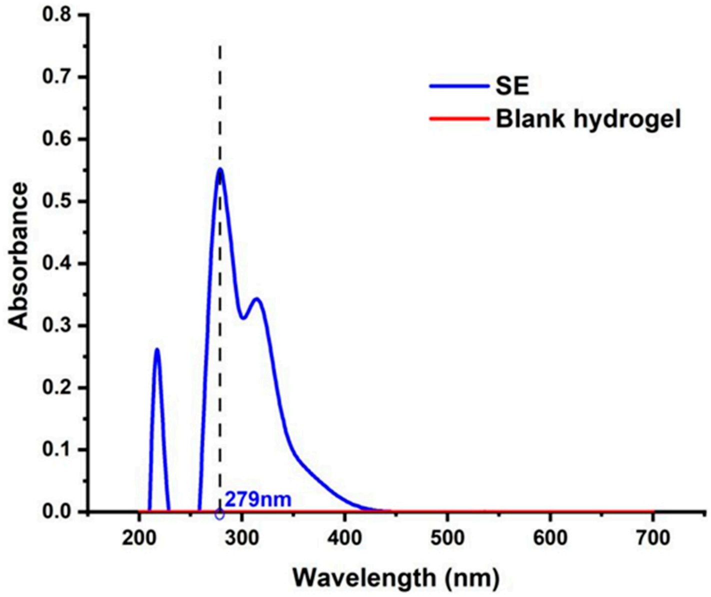

Figure S1. UV-Vis full wavelength scanning of SE and blank hydrogel. UV-Vis full wavelength scanning result of SE and blank hydrogel was shown in **Figure S1**, the biggest UV-Vis absorbance of SE is appeared in 279 nm. In the meantime, the existence of blank hydrogel had no influence on the UV-Vis absorbance of SE.

Linearity and range:

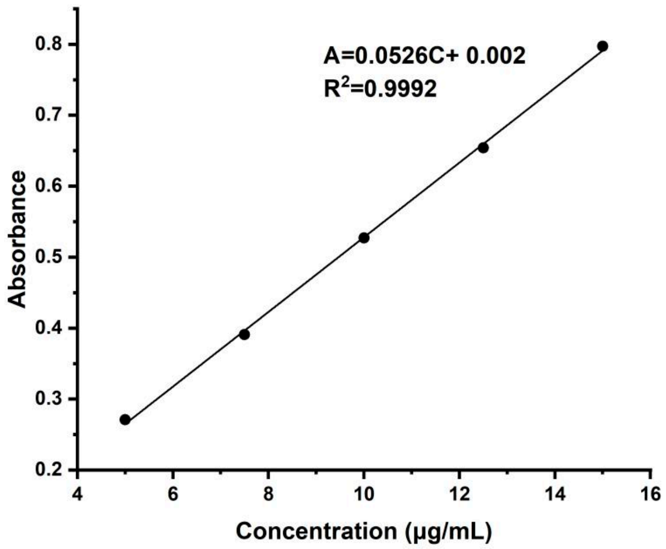

Figure S2. The standard curve of SE. SE (50 mg) was accurately weighed in a 50 mL volumetric flask. An appropriate amount of methanol was added with ultrasonic dissolution until reaching a certain volume. Upon thorough shaking, 10 mL of the solution was transferred into a 100mL volumetric flask and supplemented with an appropriate quantity of PBS solution to achieve a stock solution of 100 μg/mL. This stock solution was then systematically diluted into a series of standard solutions with concentrations of 5, 7.5, 10, 12.5, and 15 μg/mL using PBS solution. The absorbance was measured at 279 nm, with the PBS solution serving as a blank control. The resulting linear equation, obtained by conducting linear regression of the concentration C with absorbance A, is depicted in **Figure S2**.

Precision:

Instrument precision: The absorbance value of prepared standard solution (10 μg/mL) was measured at 279 nm. The assay was performed 6 times. RSD was recorded and calculated, and the results were shown in **Table S1**.

Table S1. Instrument precision results.

n	1	2	3	4	5	6	RSD (%)
Absorbance	0.527	0.528	0.529	0.530	0.527	0.531	0.31

Method precision: 1mL of stock solution was accurately measured into a 10mL volumetric flask, the PBS solution was used to stabilize volume. Steps above were operated in parallel for 6 times, the absorbance was measured at 279nm and the RSD value was calculated, and the results were shown in **Table S2**.

Table S2. Method precision results.

n	1	2	3	4	5	6	RSD (%)
Absorbance	0.537	0.525	0.532	0.539	0.549	0.536	1.48

Accuracy

SE (50mg, 100 mg, 150mg) were accurately weighed to 100mL volumetric flasks, and then the corresponding proportion of blank hydrogel was added and methanol was added to dissolve, set volume and filter. The filtrate was taken from 1mL to a 100mL volumetric flask, and the volume was fixed with PBS solution. Three concentrations were prepared (5μg/mL, 10μg/mL and 15μg/mL), which were measured sequentially at a wavelength of 279nm. The accuracy rate and RSD value were calculated. The results were shown in **Table S3**.

Table S3. Method accuracy results.

Theoretical content (μg/mL)	Actual content (μg/mL)	Accuracy (%)	Average (%)	RSD (%)
	4.98	101.01		
4.93	4.85	98.38	99.59	1.34
	4.90	99.39		
	10.17	99.80		
10.19	10.25	100.59	100.39	0.52
	10.27	100.79		
	15.38	102.19		
15.05	15.36	102.06	101.97	0.27
	15.30	101.66		

The results of the UV-Vis full wavelength scanning showed that SE has the maximum absorption at 279 nm. Based on this, a UV-Vis analysis method was established. Moreover, the linearity of the method is good in the concentration range of 5 ~ 15 μg/mL, and the precision and accuracy of the method are good.
